# Developmental Trajectories of Internally and Externally Driven Temporal Prediction

**DOI:** 10.1371/journal.pone.0135098

**Published:** 2015-08-11

**Authors:** Giovanni Mento, Vincenza Tarantino

**Affiliations:** 1 Department of General Psychology, University of Padua, Via Venezia 8, 35131, Padua, Italy; 2 Department of Neurosciences: SNPSRR, University of Padua, Via Giustiniani 5, 35128, Padova (PD), Italy; University of Groningen, NETHERLANDS

## Abstract

The ability to generate temporal prediction (TP) is fundamental to our survival since it allows us to selectively orient our attention in time in order to prioritize relevant environmental information. Studies on adult participants showed that externally and internally driven mechanisms can be engaged to establish TP, both resulting in better behavioural performance. However, few studies on children have investigated the ability to engage internally and externally driven TP, especially in relation to how these mechanisms change across development. In this study, 111 participants (88 children between six and eleven years of age, and 23 adults) were tested by means of a simple reaction time paradigm, in which temporal cueing and neutral conditions were orthogonally manipulated to induce externally and internally driven TP mechanisms, as well as an interaction between the two. Sequential effects (SEs) relative to both tasks were also investigated. Results showed that all children participating in the study were able to implement both external and internal TP in an independent fashion. However, children younger than eight years were not able to combine both strategies. Furthermore, in the temporal cueing blocks they did not show the typically-observed asymmetric SE pattern. These results suggest that children can flexibly use both external and internal TP mechanisms to optimise their behaviour, although their successful combined use develops only after eight years of age.

## Introduction

The ability to temporally predict relevant events (temporal prediction, or TP) is a fundamental skill allowing us to reduce uncertainty about the future and to optimise our behaviour [1–3]. Crossing a busy street at the rush hour, or deciding whether to accelerate or to stop when our car arrives at an amber traffic light are just two among thousands of daily situations in which a bad prediction of the correct time to act may threaten our survival. In spite of this, the ontogenesis of the cognitive mechanisms enabling human beings to develop a ‘predictive brain’ is still a challenging topic. In this study, we tried to address this issue by investigating the developmental trajectory of different types of TP.

It has been demonstrated that TP can be externally generated by the temporal properties of stimuli. More specifically, externally driven TP (E–TP) may be established by exploiting the temporal information provided either by regular sensorial structures (i.e., auditory rhythms) or discrete signals cueing the onset of task-relevant stimuli [4–15]. In both cases, the possibility of using external information to predict the onset of upcoming events induces the orienting of attention toward specific points in time. This ability is defined as temporal orienting (TO) [1,3,4]. By contrast, in the absence of external cues providing information about incoming events, TP must be deployed over time by using an internally driven process (I–TP). A classic example of I–TP is the well-known variable foreperiod (FP) effect. When FPs of different durations are randomly presented in a block of trials with the same *a priori* probability of occurrence, the mean reaction time (RT) to targets decreases with increasing FPs [16–19]. The specific benefit of long *vs*. short FP trials is mainly explained by the unidirectional flow of time itself, which intrinsically and conditionally biases target predictability [6,16,20–23]. The probability of an event occurring at a given time is conditioned by the accumulating evidence that it has not yet occurred, a phenomenon also known as hazard function [16,20–22,24]. An additional source of TP is reflected in the so-called sequential effects (SEs) [17,18,25–27]. Sequential effects rely on the observation that the temporal structure of trials itself can induce TP by biasing the allocation of cognitive and motor resources over time. Specifically, a participant’s ability to predict the onset of forthcoming events will depend on the temporal rule experienced in the preceding trial. Behaviourally, this translates into slower RTs in the current trial, when this is preceded by a longer FP in the previous trial, than when preceded by an equally long or shorter one. Notably, SEs are asymmetric, as they are mainly present with the shortest FP in a block of trials, but tend to vanish as FP increases. According to the ‘dual-process’ model proposed by Vallesi and collaborators [28–30], while SEs *per se* are automatic in nature, their observed asymmetry, depending on the relative durations, reflect a voluntary re-preparation process triggered by the conditional probability of target appearance over time. Indeed, conditional probability for target onset at the long FP is always equal to 1, irrespective of the previous FP duration.

While the cognitive mechanisms and the underlying neural bases of E–TP and I–TP in adults are relatively known [1,3,6,10,11,17,18], how they emerge and interact ontogenetically is less clear. In a pioneering study, Elliot [31] tested a large sample of children aged 5–13 years, and adults performing a simple auditory FP task, including several preparatory intervals. Unlike many following studies (see below), he only reported a slight and non-significant FP effect. However, this discrepancy may be attributed to methodological issues, including the wide ranges of intervals used (1–16 s), making it harder for children to maintain sustained attention over time. In a later study, Czudner and Rourke [32] tested children with brain-damage, subdivided into two groups, ‘young’ (6 to 9 years) and ‘old’ (10 to 13 years), by using both regular and irregular FPs. Interestingly, they found that young control, old control, and old brain-damaged groups performed better than the young brain-damaged group, suggesting that, with advancing years, brain-damaged children may adapt to and/or recover from deficit(s) in their ability to develop and maintain a state of readiness to respond. A FP effect in children between around 7 and 11 years was also reported by Ozmun et al. [33]. More recently, Vallesi and Shallice [34] investigated both FP and sequential effects in 4 to 11 year-old children, showing a developmental dissociation between the two effects; while SEs were already present at 4–5 years of age, FP appeared gradually from 6–7 years on. These findings suggest that, although being both implicit forms of temporal preparation, SEs reflect a more automatic process than variable FP effects. This suggestion is supported by the observation that only the FP, but not the SEs, are affected by dual-task interference [35–37]. In summary, the available studies provide consistent evidence that school-age children can implicitly generate TP by relying on internal sources of information; this mechanism translates into behavioural advantage in terms of motor preparation.

Concerning the ability to use E–TP, there is evidence that preverbal infants are already able to learn the temporal regularities as well as the durations associated with external sensorial events [38–42]. This suggests that the ability to exogenously orient attention in time, making use of external information, should also be operating at 6 years of age. The ability of using external, discrete cues to endogenously orient attention in time has been much less investigated in children. In a recent study, Johnson and colleagues [43] employed a cueing paradigm to disentangle the automatic and voluntary mechanisms underlying spatial and temporal orienting in children. Interestingly, they found that, although children were able to automatically orient their attention in space and time, they showed voluntary orienting of attention in space but not in time. More specifically, in the temporal cueing blocks children showed FP and SEs, but not TO effects. As discussed by the authors, the lack of significant TO in their study may be accounted for by the use of lateralised rather than central targets, which could have introduced a spatial uncertainty bias that possibly reduced the behavioural benefit provided by the temporal cues. Moreover, Johnson and colleagues did not directly test the developmental trajectories of cueing effects, given that they tested a single group of children ranging from six to sixteen years of age. Hence, the questions are still open as to whether E–TP, generated by discrete temporal cues, may emerge ontogenetically and whether and how it interacts with other forms of implicit timing involving I–TP (i.e., FP and SEs) across development.

To address the above questions, in this cross-sectional study we compared the developmental trajectories of E–TP and I–TP in six to eleven year-old children and adults according to a paradigm in which a TO and a FP task were factorially combined to respectively induce E–TP and I–TP; we also investigated their interplay. We adopted a child-friendly approach including the presentation of central targets and a fixed 100% cue validity to facilitate children in the deliberate use of cues to orient their attention in time, as well as to enhance the benefits of temporal cueing by keeping fixed spatial target predictability. Sequential effects in both TP conditions were also measured and analysed.

## Method

### Participants

One hundred and twenty participants were initially enrolled. Eight children were excluded since they did not complete the experimental task. One subject was excluded because he exceeded 2 SD from the age group RT average. The final sample was composed of 111 participants (88 children and 23 adults). Children were recruited from a primary school in the Venetian Region of Italy and were allocated into one of three groups: 6–7, 8–9 and 10–11 years. Adults were the children’s parents or undergraduate psychology students at the University of Padua. The demographic characteristics of the four groups are described in Table 1.

**Table 1:** Experimental groups. Main demographic characteristics of the participants to the study. SD = Standard Deviation.

		Gender		Handedness		
Group	Mean Age ± SD (range)	Female	Male	Left	Right	*n*
6–7	77 ± 4 months (69–84)	16	14	2	28	30
8–9	90 ± 4 months (84–96)	18	16	4	30	34
10–11	105 ± 4 months (97–127)	12	12	3	21	24
Adults	35.6 ± 15 years (18–59)	11	12	4	19	23

#### Ethics statement

All children's parents and adult participants signed a written consent form. The director of the school also signed an agreement that formally allowed the children to be tested in the school. All experimental procedures were approved by the Ethics Committee of the School of Psychology of the University of Padua (protocol n° 1362) and were conducted according to the principles expressed in the Declaration of Helsinki.

### Experimental procedure

The children underwent the experimental task individually in a quiet room at their school, while the adults completed the experimental task at the University. The same experimenter collected all behavioural data. Children reported as having learning disabilities or neurological or psychiatric disorders were excluded. All had normal or corrected-to-normal vision.

### Stimuli and tasks

Stimuli were presented on a 17–inch monitor at a resolution of 1,280 × 1,024 pixels. Participants were seated comfortably in a chair at a viewing distance of 60 cm from the monitor. All participants performed two TP tasks within the same experimental session. The trial structure was the same in both tasks.

Each trial began with the display of a visual cue, followed by the presentation of a target stimulus that stayed on the screen for a maximum of 3,000 ms. The visual cue consisted of a picture of a black camera lens surrounded by a circle (total size of the stimulus: 840 × 840 pixels, 144 dpi, 10.62° × 10.54° of visual angle). The target stimulus consisted of a picture of an animal, which was displayed centrally within the camera lens. The cue-target stimulus-onset-asynchrony (SOA) was manipulated (either 600 or 1,400 ms). The inter-trial-interval (ITI) was randomly manipulated between 600 and 1,500 ms. The task consisted of speeded target detection; participants were required to press the space bar with the index finger of the dominant hand as quickly as possible at target occurrence. To encourage participants to produce a good performance, they were given the following instruction: ‘Imagine that you are at the zoo and you have a camera. Your task is to take a picture of the animals appearing within the camera lens as soon as possible. To do that, you have to press the space bar with your index finger. Please, be careful to click as quickly as possible once you see the animals otherwise they will disappear. Please, also be careful not to click before the animals appear!’

The experimental task included temporal and neutral cueing blocks, which were administered block-wise rather than trial-by-trial in order to reduce top-down control required to switch continuously from a predictive to a non-predictive setting [36]; this may have caused additional difficulty for the children. As detailed below, children were also instructed block-by-block that they could or not predict in advance the animals’ temporal onset.

#### Temporal cueing blocks

**Fig 1:**
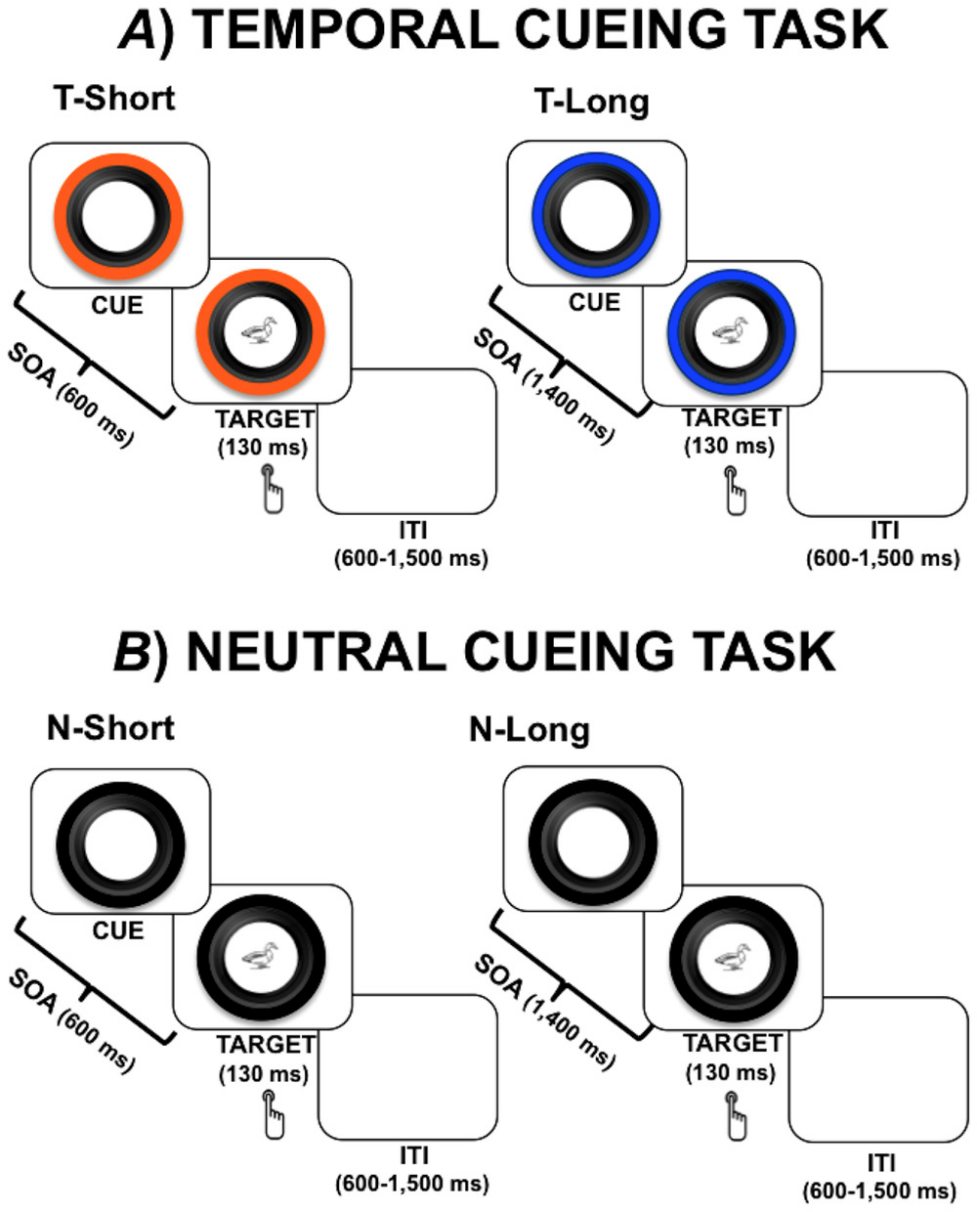
Experimental paradigm. The Temporal Cueing task (a) was purposely designed to induce externally driven temporal prediction (E–TP). The visual cue provided fixed temporal information concerning the SOA duration, which could be short (left panel) or long (right panel), according to the colour of the cue. By contrast, the Neutral Cueing task (b) was specifically designed to induce internally driven temporal prediction (I–TP). In this case participants never knew in advance the duration of the SOA, which could nevertheless have the same short or long duration than in the Temporal Cueing task.

In the temporal cueing blocks (Fig 1a) the visual cue provided fixed temporal information concerning the SOA duration. In particular, the outer circle of the camera lens was coloured either blue or orange. Each of the two colours was associated with a SOA duration (600 or 1,400 ms). In other words, the colour of the visual cue provided fixed information about the SOA duration, leading to a temporal short (T–Short) and a temporal long (T–Long) SOA condition. The association between the colour of the outer circle and the SOA duration was counterbalanced between subjects. In order to maximise the E–TP magnitude, the cue was 100% valid (the association between the colour and the SOA remained fixed throughout the task), no catch trials were included (the target stimulus always appeared after the SOA) and the target was always presented centrally.

#### Neutral cueing blocks

In the neutral cueing blocks (Fig 1b), the outer circle surrounding the camera lens was always black, providing no information about SOA duration; the cue simply acted as a warning signal, which prepared participants for the forthcoming target without furnishing temporal information about it. Nevertheless, regarding the temporal cueing blocks, the SOA was also manipulated to create a neutral short (N–Short) and a neutral long (N–Long) SOA condition (600 and 1,400 ms, respectively). To maximise the magnitude of the I–TP effect, we used an ‘aging’ probability distribution [16,44], with an equal *a priori* odds ratio for each SOA duration. This probability distribution is known to obey a hazard function [6,16,20]; that is, the subjective probability of target onset will increase over time, given that it has not yet occurred.

### Experimental design

**Fig 2:**
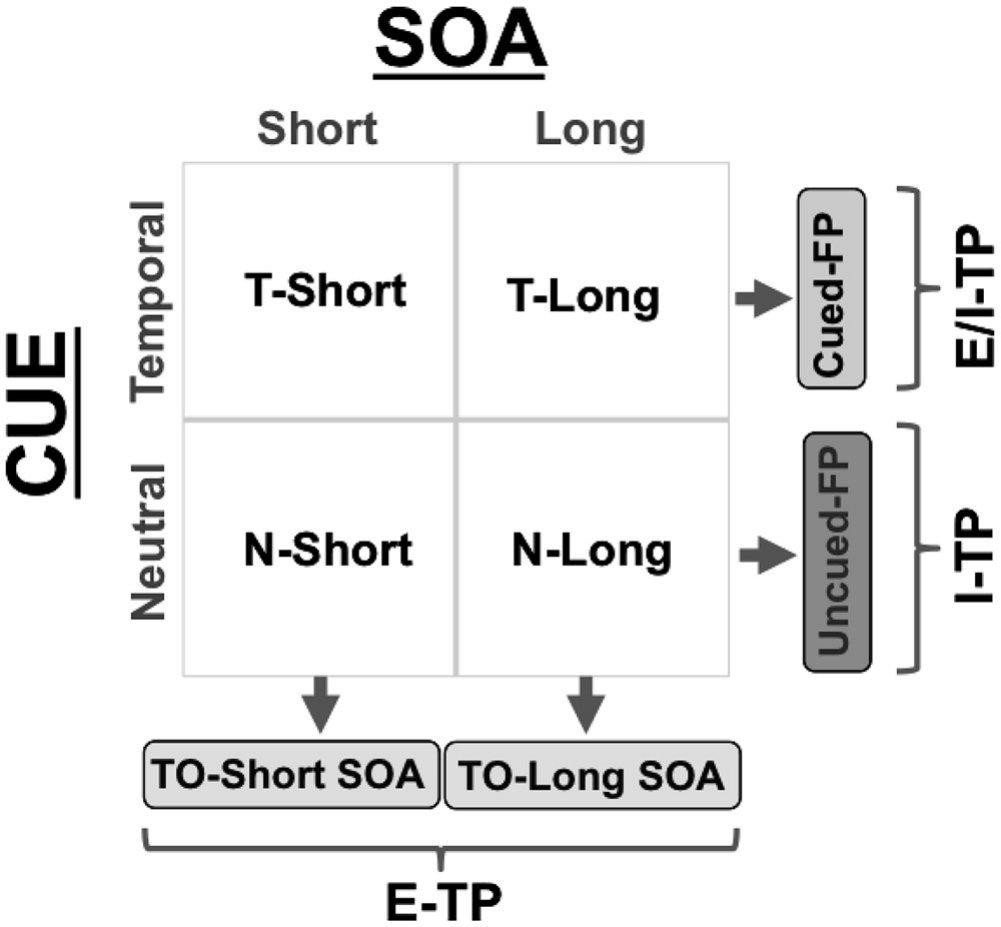
Experimental Design. The cue (Temporal *vs*. Neutral) and the SOA (Short *vs*. Long) were factorially combined to obtain a measure of E–TP (revealed as the TO effect at short and long SOA), I–TP (revealed as the Uncued–FP effect) and E/I–TP (revealed as the Cued–FP effect).

As illustrated in Fig 2, the experimental manipulation yielded a factorial design in which the CUE (temporal *vs*. neutral) and the SOA (short *vs*. long) factors were orthogonally contrasted to calculate E–TP, I–TP and their combined effect (E/I–TP) on behavioural performance. Specifically, the E–TP effect was measured by comparing mean RTs between N–Short and T–Short trials (*TO–Short SOA*) and between N–Long and T–Long trials (*TO–Long SOA*). The effect of I–TP was measured by comparing mean RTs in N-Short trials to mean RTs in N–Long trials (*Uncued–FP Effect*). Finally, the combined effect of E- and I–TP was measured by comparing mean RTs in T–Short trials to mean RTs in T–Long trials (*Cued–FP Effect*). In the latter case, the performance of participants is supposed to be influenced by both the temporal information of the cue (always valid) and the hazard function (i.e., the elapsing of time itself), resulting in an interaction of external and internal TP (E/I–TP). Notably, all conditions were matched for sensorimotor requirements, since the visual stimuli and the required response were always the same across the experiment; the only difference between conditions was the level of target predictability.

Before starting the experimental session, participants were presented with a block of 20 training trials for each condition, to ensure they understood task instructions and had learnt the temporal association between cue–type and SOA in the temporal cueing task. After training, the participants were asked to identify the temporal interval associated with each of the cues to ensure understanding of the cues, and were reminded to detect the targets as quickly as possible. All the children were able to correctly identify the cue–SOA association before starting the experimental blocks. After concluding the training, each child underwent a total of four experimental blocks, including two temporal cueing and two neutral cueing blocks. Each block lasted approximately four minutes and participants were able to take breaks between the blocks. For all conditions, a total of 40 trials were delivered. The experiment was block-wise randomised. The order of the tasks was counterbalanced between subjects. E-prime 2 software (Psychology Software Tools, Pittsburgh, USA) was used to create and administer the stimuli.

### Data analysis

As independent variables, age group (6–7 years *vs*. 8–9 *vs*. years *vs*. 10–11 years *vs*. adults), cue (temporal *vs*. neutral), and SOA duration (short *vs*. long) were considered. Mean accuracy and mean RTs were the dependent variables. Omissions, anticipated responses (within the cue and 100 ms after target onset), and delayed responses (1,500 ms after target onset) were considered errors and excluded from analysis. Response times exceeding 2 SD from the individual average for each dependent variable and for each participant were excluded. The remaining responses were considered correct. Moreover, participants showing mean RTs deviating 2SD or more from the group average were excluded. The Kolmogorov-Smirnov test revealed that accuracy scores (percentages of correct responses) were not normally distributed. Hence, non–parametric tests were applied for accuracy analyses. Linear mixed models were conducted to test for between– and within–subject effects on RTs since these were normally distributed. To reliably control for the possible age–related effect on behavioural performance, RT raw data were logarithmically transformed. As suggested by Puccioni and Vallesi [45], this allows the reduction of intra– and inter–subject variability by partialling out the age-related effect across conditions. Hence, significant statistical effects resisting logarithmic transformations can be considered as being due to reliable condition–specific effects rather than to the effects of general factors, such as age–related motor response speed [46].

To explore the developmental trajectories of E–TP and I–TP and their combined effect (E/I–TP) on behavioural performance, a 2 (Cue) × 2 (SOA) × 4 (Group) mixed ANOVA was performed. Specifically, the behavioural benefit conferred by E–TP is revealed by the main effect of Cue while its developmental trajectory was measured by the Cue × Group interaction. The behavioural benefit conferred by I–TP is revealed by the main effect of SOA, and its developmental trajectory by the SOA × Group interaction. Finally, E/I–TP was tested by the Cue × SOA interaction, and its developmental trajectory by the 3-way Cue × SOA × Group interaction. To evaluate the difference in the magnitude of the single TP effects across groups, planned comparisons (one-way ANOVAs) were performed contrasting RTs on T–Short *vs*. N–Short; T–Long *vs*. N–Long; N–Short *vs*. N–Long; and T–Short *vs*. T–Long. In addition, Pearson’s correlations were performed only on the children’s data to assess the relationships between age and RTs for all TP effects (TO–Short, TO–Long, Uncued–FP and Cued–FP).

Sequential effects were tested by comparing mean RTs in the current trial (SOA_n_) to those obtained in the preceding trial (SOA_n-1_) across groups for both temporal and neutral cueing tasks. More specifically, a 2 (Cue) × 2 (SOA_n_) × 2 (SOA_n-1_) × 4 (Group) mixed ANOVA was performed. The overall behavioural benefit of SEs was revealed as the behavioural benefit conferred by preceding trials on the current ones (main effect of SOA_n-1_), and its developmental trajectory by the SOA_n-1_ × Group interaction. The Cue × SOA_n-1_ interaction tested SEs in relation to the task (temporal *vs*. neutral cueing), while its developmental trajectory was revealed by the Cue × SOA_n-1_ × Group interaction. The SE asymmetry was globally tested by the SOA_n_ × SOA_n-1_ interaction, and its developmental trajectory by the 3–way SOA_n_ × SOA_n-1_ × Group interaction. Finally, the SE asymmetry in relation to the task (temporal *vs*. neutral cueing) was revealed by the 3–way Cue × SOA_n_ × SOA_n-1_ interaction, and how it is influenced by age by the 4–way Cue × SOA_n_ × SOA_n-1_ × Group interaction. For all analyses, an alpha level of 0.05 was used. Post-hoc Fisher's least significant difference (LSD) comparisons were performed to evaluate pairwise differences among the means. The effect size was estimated by the partial eta square measure (η_p_^2^). When appropriate, critical values were adjusted using the Greenhouse–Geisser correction for violation of the assumption of sphericity.

The raw response time data from the experiment are available in S1 Dataset.

## Results

Accuracy and RT scores are reported in Table 2.

**Table 2:** Behavioural measures. Mean and standard deviation (in parentheses) measures of accuracy (percentage of correct responses) and speed (reaction times) for all groups and conditions.

	Accuracy (%)				Mean RTs (ms)			
Group	T-Short	T-Long	N-Short	N-Long	T-Short	T-Long	N-Short	N-Long
**6–7**	93.4(1.1)	91.5(1.7)	95.7(1)	92.2(1.4)	490(12)	491(12)	562(15)	501(13)
**8–9**	94.5(1.8)	96.7(0.9)	98.7(2)	96.5(0.9)	433(13)	401(10)	481(12)	422(11)
**10–11**	95.2(0.9)	96.6(0.9)	98.6(0.5)	96.8(0.7)	390(11)	359(11)	433(13)	379(12)
**Adults**	95.7(1.1)	95.6(1.7)	99(0.5)	94.5(1.8)	313(12)	291(8)	355(10)	306(10)

Note that the analyses of variance reported in the text were performed using RT log-transformed data whereas the in the table we report raw RT data for reasons of clarity.

Overall, delayed and null responses decreased as a function of age; however, significant differences emerged only in the T–Long condition (Kruskal-Wallis test H (3, N = 111) = 13.2, p < 0.01), where the 6 to 7 year-old children showed a significantly lower accuracy level (M = 91.5, SD = 1.34) compared to older children and adults.

**Fig 3:**
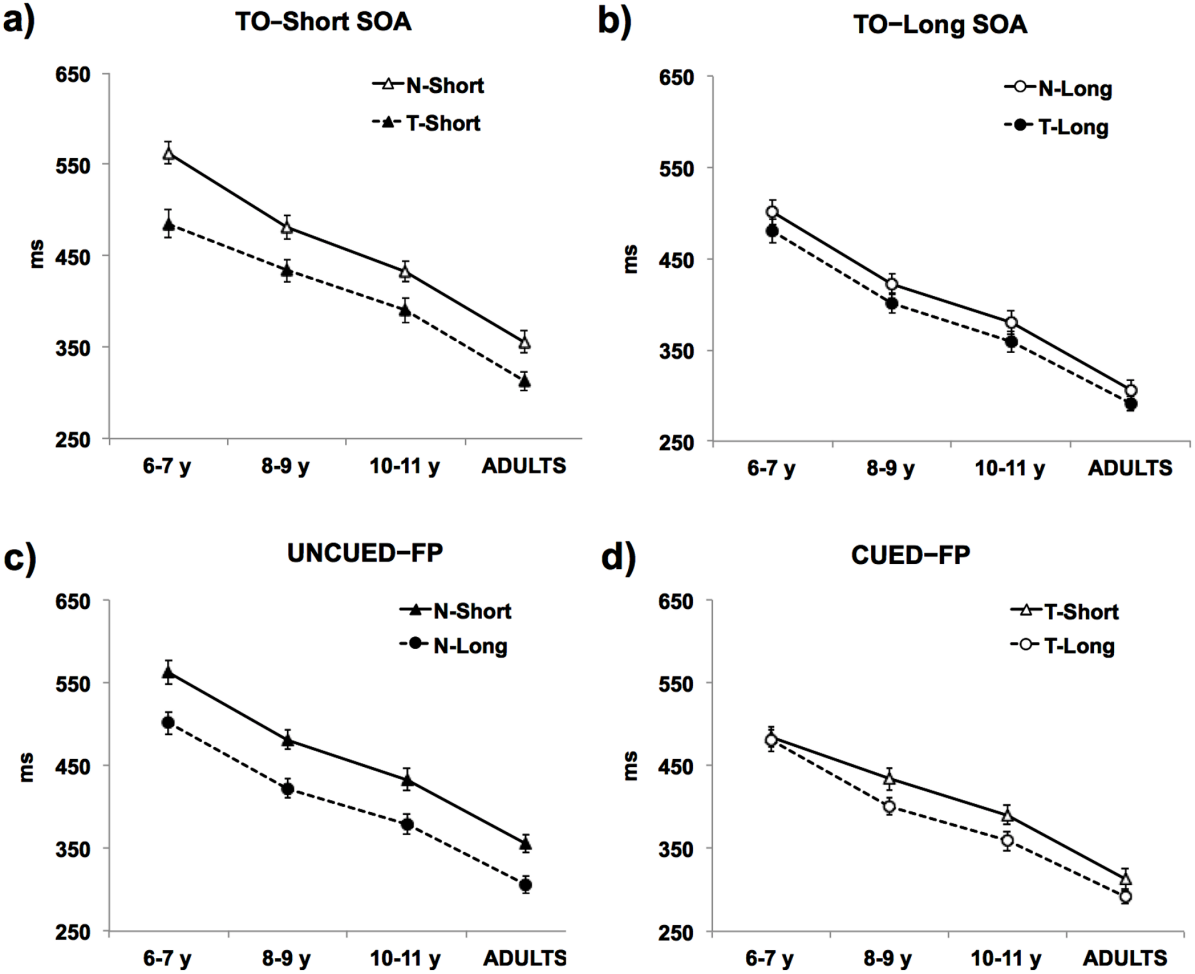
Mean reaction times. Panels show the pair-wise comparison of the mean reaction times for the TO–Short (a), TO–Long (b), Uncued–FP (c) and Cued–FP (d) effects for each age group. Error bars indicate standard errors of the mean.

Mean RTs for each TP effect and Group are plotted in Fig 3. The 2 × 2 × 4 mixed ANOVA revealed an expected Group effect (*F*(3,107) = 51.1, *p* < 0.0001, η_p_^2^ = 0.59). Post-hoc tests confirmed that RTs were progressively faster with age (6–7 years > 8–9 years > 10–11 years > adult RTs, all *p*_*s*_ ≤ 0.04). The ANOVA also revealed the presence of the TO effect (main effect of Cue; F(1, 107) = 92.33, *p* < 0.0001, η_p_^2^ = 0.46), with participants being faster in detecting temporally than neutrally cued targets (Fig 3a and 3b), as well as the presence of the FP effect (main effect of SOA; F(1, 107) = 60.89; *p* < 0.001, η_p_^2^ = 0.36), with RTs significantly shorter in long than short SOA trials (Fig 3c).

The Cue factor did not interact with Group ((F(3, 107) = 0.82; *p* > 0.95; η_p_^2^ = 0.02), while a significant two-way Cue × SOA interaction emerged (F(1, 107) = 60.89; *p* < 0.001; η_p_^2^ = 0.36). Post-hoc tests revealed all comparisons to be significant, although the behavioural benefit conferred from temporal cues was greater in short (mean TO effect = 51 ms; Cohen’s d = 0.53) than long trials (mean TO effect = 16 ms; Cohen’s d = 0.17) SOAs (p < 0.001). This asymmetric effect was explained by the mean RTs in the T-Long trials being significantly faster than the mean RTs in the T-Short trials (t(110) = -5.44; p < 0.001) and in N-Long (t(110) = -4.74; p < 0.001).

A significant interaction between Group and SOA was also found (*F*(3, 107) = 3.93; *p* < 0.02, η_p_^2^ = 0.09). Planned comparisons confirmed that, when considering together temporal and neutral cueing conditions, the SOA effect (faster RTs for long than short SOAs) emerged gradually, with the younger children showing a smaller effect (mean SOA effect = 29 ms; Cohen’s *d* = 0.22) than older children and adults (all mean SOA effects ≥ 35 ms; all Cohen’s *ds* ≥ 0.37).

**Fig 4:**
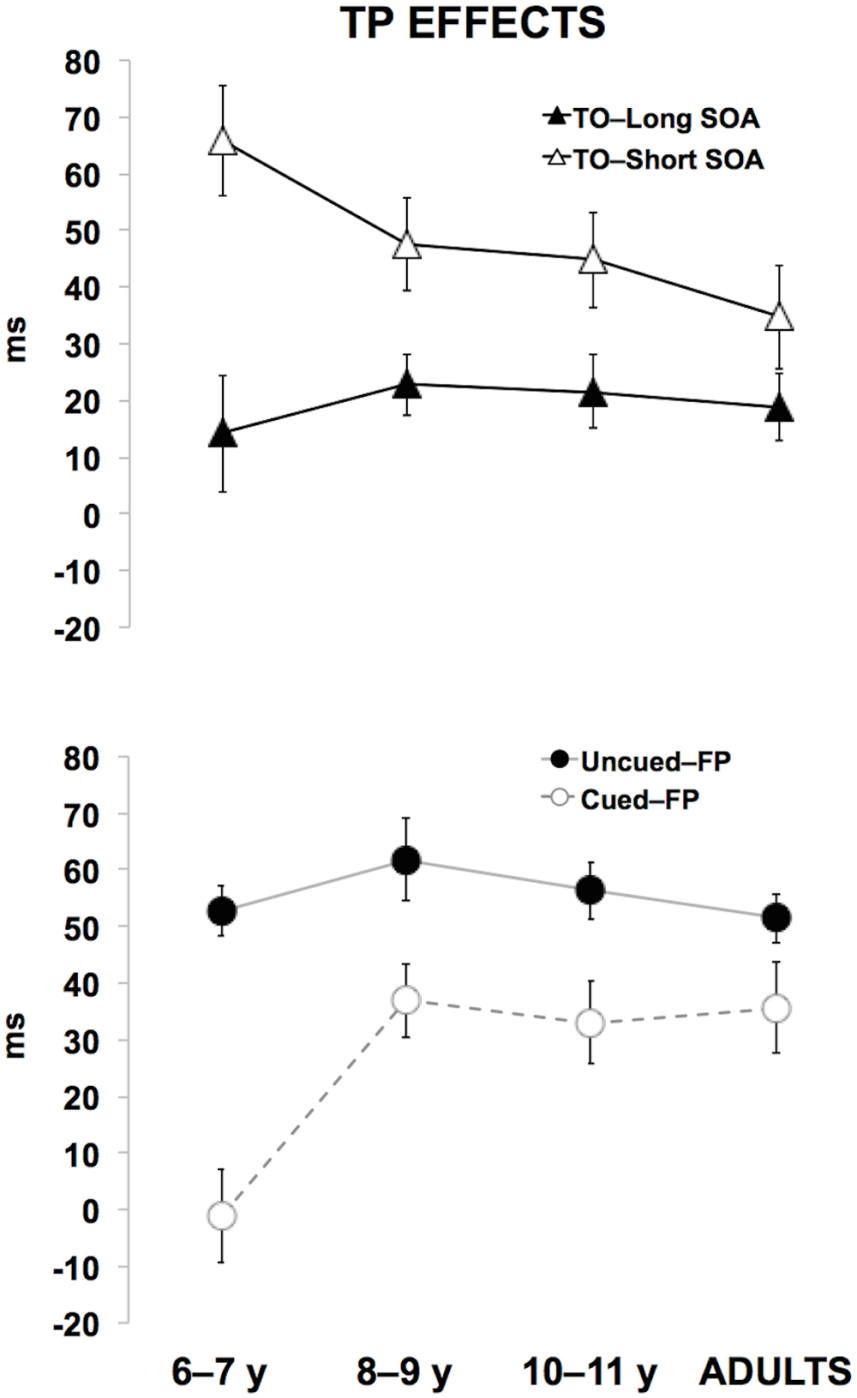
Developmental trajectories of each TP effect. The upper panel shows the developmental trajectories of each single TP effects derived by pair-wise comparisons. In particular, the upper panel shows TO–Short (T–Short minus N–Short) and TO–Long (T–Long minus N–Long) effects, both measures of E–TP. The lower panel displays the developmental trajectories of the Uncued–FP (N–Long minus N–Short) effect as a measure of I–TP and of the Cued–FP (T–Long minus T–Short) effect as a E/I–TP combined measure. Error bars indicate standard errors of the mean. Note that the analyses of variance reported in the text were performed using log-transformed data whereas the the figure shows raw data for reasons of clarity.

Most importantly, we found a significant 3-way Cue × SOA × Group interaction (*F*(3, 107) = 2.79; *p* < 0.05, η_p_^2^ = 0.07; Fig 3d). To better identify the source of this interaction, planned comparisons across age groups (one-way ANOVAs) were performed. These allowed to statistically test for the presence of possible developmental dissociations for each TP effect. One-way ANOVAs revealed no group effects on the TO–Short SOA, the TO–Long SOA or the Uncued–FP effects (all F_s_(3, 107) < 1.18; all p_s_ > 0.32). By contrast, the Cued–FP effect was significantly affected by age (F(3, 107) = 4.64; p < 0.005, η_p_^2^ = 0.14). The post-hoc analysis revealed that 6 to 7 year-old children significantly differed from older groups (all p_s_ ≤ 0.02). More specifically, as depicted in Fig 4, the Cued-FP effect was absent (around zero) in the youngest group, but present and stable at later ages. In other words, the combined effect of internally and externally driven TP (E/I–TP) emerged after 8 years of age.

### Sequential effects

The 4 × 2 × 2 × 2 mixed ANOVA yielded a main SOA_n-1_ effect (*F*(1, 107) = 194.31; *p* < 0.0001, η_p_^2^ = 0.64), confirming that RTs in the current trials were affected by the preceding ones (SEs). This effect was further influenced by development, as shown by the SOA_n-1_ × Group interaction (*F*(3, 107) = 2.92; *p* < 0.05, η_p_^2^ = 0.07). This interaction was explained by the observation that the advantage provided by the preceding trial on the current one diminished as age increased, being greatest for the youngest group (43 ms on average) and smallest for the oldest one (15 ms on average).

We also found a SOA_n_ × SOA_n-1_ interaction (*F*(1, 107) = 64.7; *p* < 0.001, η_p_^2^ = 0.38). This interaction was explained by the fact that SEs were asymmetric, that is, RTs were overall shorter on the current trials when these were preceded by short rather than long SOA trials. The ANOVA further revealed a 3–way Cue × SOA_n_ × SOA_n-1_ interaction (*F*(3, 107) = 14.91; *p* < 0.001, η_p_^2^ = 0.17). This interaction revealed that, in the temporal cueing condition, the SEs were less asymmetric than in the neutral cueing condition.

**Fig 5:**
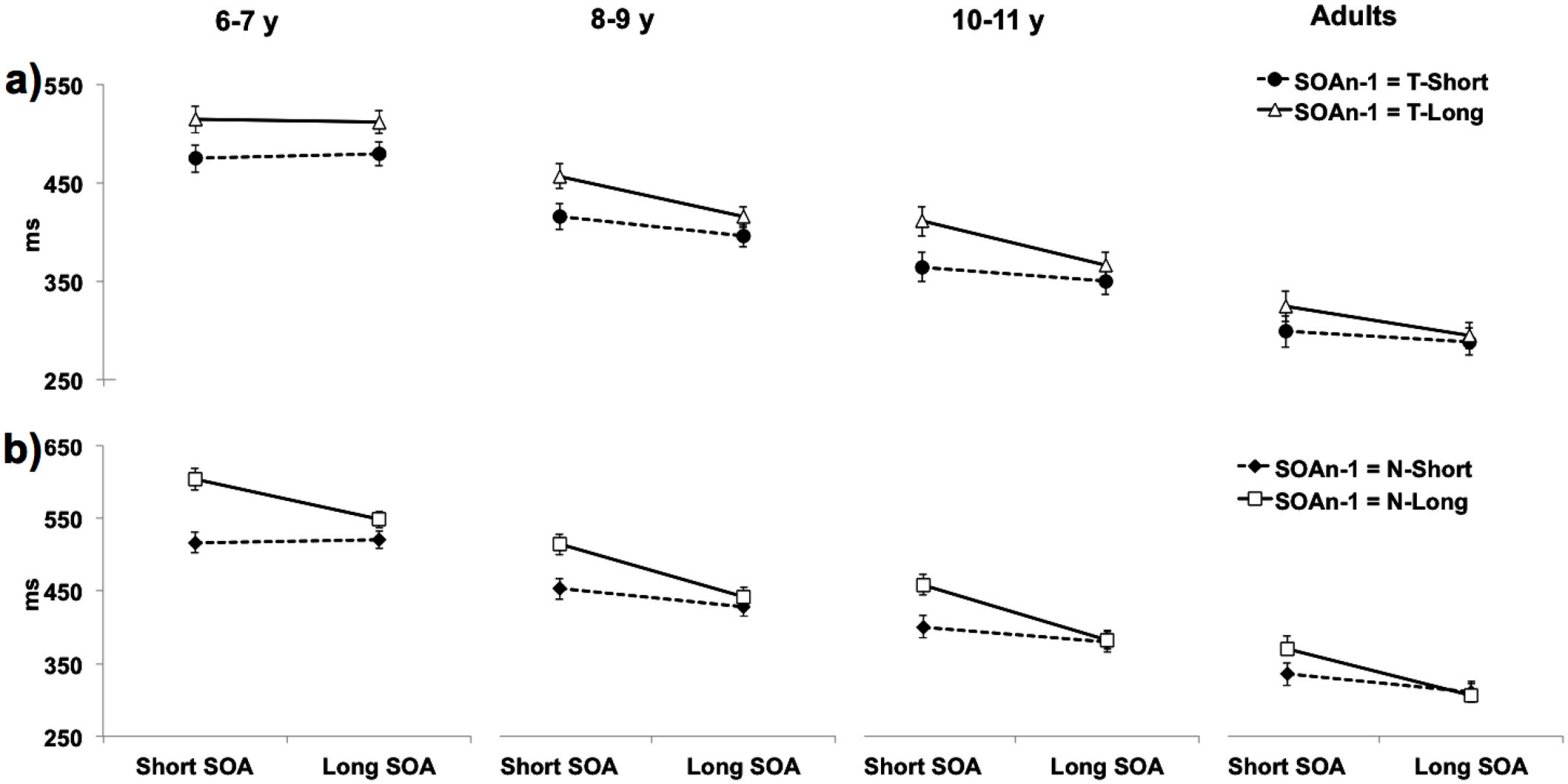
Sequential Effects. Mean RTs are plotted as a function of the current (SOA_n_; x axis) and preceding (SOA_n-1_; lines) cue-target intervals for each age group and for Temporal (a) and Neutral (b) cueing Tasks. Error bars indicate standard errors of the mean. Note that the analyses of variance reported in the text were performed using log-transformed data whereas the the figure shows raw data for reasons of clarity.

The 4–way Cue × SOA_n_ × SOA_n-1_ × Group interaction was not significant (*F*(3, 107) = 0.13; *p* > 0.94, η_p_^2^ = 0.01). It should be noted that a lack of significant interaction between SE asymmetry and age group was previously reported by Vallesi and Shallice [34], who tested 4 to 12 year-old children with a variable FP paradigm. As discussed by these authors, this null interaction effect may be accounted for by the high intra–and inter–subject variability of RTs in children that could be only reduced, but not completely eliminated, by log-transformation [46]. Although the 4-way interaction was not significant, the visual inspection of the developmental trajectory of each TP effect plotted separately for cueing conditions (Fig 5) suggests that SEs shown by 6 to 7 year-olds in the temporal cueing task were symmetrical as compared to those seen in the neutral cueing task and older children.

Following Vallesi and Shallice [34] statistical approach, to corroborate this observation subsequent 2 (Cue) × 2 (SOA_n_) × 2 (SOA_n-1_) repeated-measures ANOVAs were performed separately on each group, with SOA_n_ and SOA_n-1_ as the within-subjects variables. It emerged that the Cue × SOAn × SOAn-1 interaction was significant only in the 6 to 7 year–old group (F(1, 29) = 4.72, p < 0.05) but not in the older ones (F(1, 33) = 2.26 for 8–9 year olds; F(1, 23) = 3.63 for 10–11 year olds; F(1, 22) = 3.44 for adults). Post-hoc tests conducted on the 6–7 year-old group confirmed that, in the temporal cueing task RTs in the current trials were equally affected when preceded by short or long SOA_n-1_ intervals (both p < 0.01). Whereas, in the neutral cueing task the SE was present in short SOA_n_ (p < 0.001) but not in long SOA_n_ trials (p > 0.07). In other words, they showed symmetrical SEs. By contrast, in the neutral cueing task SEs were present only in short SOA_n_ trials, with all participants being faster when these were preceded by short rather than long SOA_n-1_ trials (symmetrical SEs).

### Correlational analyses

The correlational analyses conducted between the age and each single TP effect revealed that the only index showing a significant correlation with age was the Cued–FP effect (r = 0.43, 95% BCa CI [.27, .59], Bonferroni adjusted-p < 0.02), while the other effects were not affected by age (all rs < 0.17; all ps > 0.07). That is, while the behavioural benefit conveyed separately by E–TP and I–TP was developmentally stable, the effect conferred by their combined use increased significantly with age.

## Discussion

The main aim of the present study was to investigate the developmental trajectories of different types of temporal prediction (TP) mechanisms in typically developing school-aged children. To our knowledge, this study is the first purposely designed to delineate the developmental trends of internal (i.e., the information provided by the passage of time itself) *vs*. external (i.e., the temporal information provided by discrete cues) TP mechanisms in such population. Overall, the results demonstrate that all children participating at the study (from six to eleven years) were able to implement internally (E–TP) and externally (I–TP) driven TP mechanisms, showing an efficient ability to orient attention in time and, consequently, to optimise their behavioural performance. The ability to implement E–TP was demonstrated by the observation that children benefitted from the information provided by external discrete temporal cues to improve their performance in terms of target detection speed (i.e., TO effect). Furthermore, findings highlighted two noteworthy issues. First, the TO effect did not interact with age, following a stable developmental trajectory. Indeed, while RTs showed a linear shortening with increasing age, the RT difference between temporally *vs*. neutrally cued targets remained nearly constant across age. Second, the presence of this effect was independent from the SOA duration, although its magnitude was larger for short than long SOAs. In other words, all participants exhibited faster RTs in temporal cueing as compared to neutral cueing blocks for both short and long SOA durations, suggesting that the cognitive mechanisms underlying E-TP generated by discrete cues are fully established as early as six years of age and do not show significant changes over time.

A recent study by Johnson and colleagues [43] employed a cueing paradigm to test the mechanisms underlying the ability to orient attention in space and time in children. Interestingly, they found that children can voluntarily and automatically orient attention in space, as revealed by the presence of both cueing and sequential effects on children’s behavioural performance. However, in contrast to our findings, they found only automatic, but not voluntary, temporal orienting in children. That is, children did not benefit from the temporal information provided by symbolic cues (TO effect) although their performance was affected by the conditional probability of target occurrence in time (FP effect) as well as the sequential temporal structure of trials (SEs). There are several reasons possibly accounting for the lack of consistence between the results of Johnson and colleagues and those we report in the present study. Firstly, as discussed by the authors, the target presentation in their paradigm was lateralised rather than central; this experimental choice may have disrupted the cueing effect in children. Indeed, it has been shown that the benefit of temporal cues is enhanced when the target position is fixed and therefore spatially predictable [47]. Hence, it is possible that in our study the central presentation of target stimuli and the consequent fixed spatial focus of attention made it easier for children to benefit from temporal cues. A second possible explanation may rely on the difference in the degree of temporal certainty experienced by children in the two studies. While Johnson and colleagues manipulated the cue validity (i.e., 80% of the cues provided valid, and 20% invalid, information), we kept the validity of the cue constant to 100%. It follows that, in order to optimise behaviour, in our study children could benefit both from the temporal cue and from the maximum temporal certainty that the target would have appeared always at the time predicted by the cue. By contrast, the invalid conditions used by Johnson and colleagues may have introduced an additional difficulty given that, in such a case, children could not completely trust the temporal information provided by the cue. It is also possible that the mixing of temporal and neutral cueing conditions within the same experimental block in Johnson et al.’s study may have made the task too challenging for children (especially for the younger ones). In line with this, in a pilot study (unpublished data), we found that when using an event-related design including both temporal and neutral cues in the same block, children failed to show TO effect, even if the target was centrally presented. We suggest that memory demands associated with maintaining, retrieving and updating the cue meaning from trial to trial, as well as the necessity to switch from a predictive to a non predictive context, may have selectively impaired temporal, but not spatial, voluntary orienting. As suggested by the authors, it should be taken into account that using cues to orient attention in time may be conceptually more demanding than using spatial cues.

Finally, we cannot completely exclude the possibility that the different kinds of stimuli employed in the two studies may have further affected the possibility of eliciting the TO effect in children. Indeed, in our study, we used child-friendly stimuli (i.e., coloured circles and pictures of animals for the cue and the target, respectively) while Johnson and colleagues presented children with symbolic stimuli representing different parts of a clock-face for the cue and the ‘+’ or ‘x’ signs for the target stimuli, which may be more difficult to discriminate. Most importantly, Johnson and colleagues tested a single group of six to sixteen year-old children, whereas in our study children were younger than eleven years. From eleven to sixteen years, neurodevelopmental outcomes substantially change. In summary, in the light of the above considerations it is difficult to draw reliable conclusions from the comparisons of the two studies.

A second finding of our study was that children were able to use internal information (i.e., the passage of time itself) to predict target occurrence and consequently to orient attention in time (I–TP). According to our experimental paradigm, the I–TP could be measured as the benefit in RT speeding up, conferred by long *vs*. short SOAs in the context of either the temporal (Cued–FP) or the neutral (Uncued–FP) cueing task. All participants showed a global shortening of RTs as well as a reduction of E–TP effect in long SOAs due to the variable FP effect [13–17]. The FP effect is generally explained by the hazard function [6,16,26], which is defined as the conditional probability of an event occurring at a given time, given that it has not occurred yet. In other words, the boost of expectation conditionally induced from the passage of time heightened the probability of target occurrence, inducing faster responses in long cue–target intervals. Similarly to TO, in the neutral cueing task the Uncued–FP effect did not show any interaction with age, suggesting that the mechanisms underlying I–TP are already developed and functioning at six years of age. This evidence is in line with most of previous studies reporting FP effects in children [32–34,43, 48–49].

As a third important finding, we found that, although even young children were able to independently use internal and external mechanisms to generate TP, and consequently to orient their attention in time, their ability to combine these two sources of information emerged only after eight years of age. According to our experimental design, the Cued–FP effect measures the combined use of external and internal TP processes. Namely, in the temporal cueing blocks, the occurrence of targets is highly predictable based on the full validity of the cue (TO effect) as well as on the hazard function that conditionally biases target predictability (FP effect). Hence, the combined effect of both these sources of information on target occurrence leads to the highest level of target expectancy, and therefore to the lowest RTs in T–Long trials. The evidence that in the temporal cueing blocks the FP effect (Cued–FP effect) emerged only after eight years of age demonstrates that the ability to make a combined use of E–TP and I–TP is developmentally constrained.

We found a developmental dissociation also in SEs; in all age groups the behavioural performance in the current trials (SOA_n_) was overall affected by the duration of the preceding ones (SOA_n-1_). Yet, the magnitude of this effect depended on the SOA_n_ duration; that is, the SEs were greater in short rather than long SOA_n_, confirming the typical asymmetrical pattern reported in the literature [17,18,26]. Critically, as suggested by the visual inspection of mean RTs (Fig 5), the SE asymmetrical pattern seemed to be further affected by the interplay between age group and task. Namely, in the temporal cueing task, the behavioural advantage conferred by the preceding trials on the current ones appeared to be comparable for short and long SOA_n_ (i.e., SE symmetrical pattern) only in the six to seven-year old children, while this difference became biased toward the short SOA_n_ (i.e., asymmetrical SE pattern) at later ages. By contrast, in the neutral cueing task, the magnitude of the SE asymmetry appeared to be constant across all groups. Yet, we did not find an ominibus statistical validation of this effect, since the ANOVA did not yield a significant 4-way interaction. As suggested by Vallesi and Shallice [34], the lack of significant interaction between age, task and the emerging of the typical SE asymmetry can be accounted for by the high inter– and intra–subject variability, that can be only reduced, but not completely eliminated, after applying a logarithmic transformation on raw RTs [46]. Although the null 4-way interaction represents a limitation of the present study, we nevertheless tried to provide further statistical evidence in support of the observed data. To this aim, we conducted additional 3-way ANOVAs separately for the temporal and neutral cueing task. These confirmed that, in the youngest children, the SE pattern was symmetrical only in the temporal cueing task, given that their behavioural performance in SOA_n_ trials was equally affected irrespective of the duration of the preceding SOA_n-1_ trials. Remarkably, in the neutral cueing task all groups, including the youngest one, exhibited the typical asymmetric pattern described in the literature, with RTs in the current short, but not long, trials being affected by SOA_n-1_ durations.

The apparent presence of a developmental dissociation including either the Cued–FP effect and the symmetrical SEs demonstrates that, while from six years of age onwards children can successfully and independently make use of external and internal TP mechanisms to orient attention in time, the ability to combine these two different sources of information emerges only from eight years of age. A possible explanation for this is that, before eight years of age, their limited available cognitive resources do not allow children to make a combined use of external and internal strategies. From a theoretical perspective, according to the dual process view [18] a controlled monitoring of the change of conditional probability would explain both the FP effect and the asymmetry of SEs. In the first case, the subjective target predictability will be highest in the longest SOAs due to the hazard function. In the second case, in any trial the participant implicitly expects a repetition of the previous SOA, hence, the maximum preparedness is biased toward the same SOA as that of the previous trial. If SOA_n_ is shorter than SOA_n-1_, then participants will be not fully prepared when the imperative stimulus occurs, resulting in relatively slower RTs. However, when SOA_n_ is longer than SOA_n-1_, it is assumed that participants can update and re-orient their attention over time and consequently re-prepare for optimal preparation, resulting in SE abolishment for long intervals. Within this theoretical framework, we speculated that, for young children, it may be difficult to efficiently monitor the time–based conditional probability of target onset while concurrently using the temporal information provided by the cues, which implies that the Cue–SOA associations has to be learnt, maintained and retrieved trial-by-trial. This hypothesis is corroborated by the observation that only the SE asymmetry, but not the presence of SEs *per se* (which is assumed to be more automatic by the ‘dual process’ view), was disrupted in young children. In other words, the effort made in using external, discrete cues to generate predictions over time may have disrupted the monitoring of the conditional probability of target occurrence in young children.

As an alternative explanation, we propose that six to seven year-old children may not yet have developed the capacity to disengage attention from externally to internally driven information when the two have to be simultaneously processed, requiring considerable effort to re-orient attention over time in long SOA_n_ trials. In fact, younger children can effortlessly disengage and re-orient attention in the current trials (FP effect) as well as from the duration of the preceding trial (SE asymmetry) when they only need to use I-TP. However, when they must make a combined use of I–TP and E–TP, the attentional disengagement may result more effortful for them, as revealed by the absence of both the Cued–FP effect and by the SE asymmetry only in temporal cueing blocks. In support of this hypothesis, we also found that the Cued–FP effect, which is assumed to depend on attentional resources more than other TP measures, is the only index showing a positive correlation with age. That is, the older the child, the stronger the Cued–FP.

A further, simpler hypothesis explaining why younger children did not show FP effect and SE asymmetry in the temporal cueing task, is also that at this age children invest considerable attentional resources on externally available cues, given that this is the first useful information always providing valid target predictability. In other words, they can immediately and completely use the cues to speed up their RTs to targets. By contrast, in the case of I–TP, children are only required to perceive the elapsing of time, given that the cues do not provide useful information to generate predictions. Then, they have to use the internal information to conditionally prepare for the next time in which targets are likely to occur. Even if young children can effortlessly build up a mental representation of durations, as indirectly shown by the presence of Uncued–FP effect, this operation could be more demanding for them when it must be combined with the processing of information externally provided by discrete cues, which do not require *per se* a dynamic and metric temporal representation. Though, further studies are needed to better disentangle the above different accounts. A possible way to better unravel the developmental mechanisms underlying dissociations in TP effects may come from neurophysiological studies depicting the timing of the neural mechanisms underlying I–TP and E–TP across development. Although our present data do not allow neurophysiological hypotheses to be tested directly, a possible hypothesis to be addressed may be that while the single cortical networks underlying E–TP and I–TP are already established at six years of age, they cannot be simultaneously recruited before the age of eight. It may be also possible that their functional connectivity may not be yet developed until this time. In support of this view, structural neuroimaging studies using diffusion tensor imaging (DTI) demonstrated a developmental increase in grey matter activation in frontal and parietal cortices which would be in turn the direct consequence of the maturation of white matter connection [50]. More specifically, the inferior parietal cortices, which have been shown to play a key role in temporal orienting [3,4,23,51–54] and the lateral pre-frontal cortices, which are implied in the monitoring of the hazard function [5,6,18,21] are among the last areas to myelinate, a change that would result in more stable fronto-parietal activity. Although the neuroimaging approach promises to provide a useful key for a deeper understanding of the developmental mechanisms underlying TP phenomena in children, much work has yet to be done in this field.

## Supporting Information

S1 Dataset Mean accuracy and response time for each participant. (XLSX)
